# Comparative Outcomes of Primary Arterial Switch Operation for Transposition of Great Arteries within the First Month of Life

**Published:** 2020-01

**Authors:** Hamid Bigdelian, Mohsen Sedighi

**Affiliations:** 1 *Department of Cardiovascular Surgery, School of Medicine, Isfahan University of Medical Sciences, Isfahan, Iran.*; 2 *Department of Cardiovascular Surgery* *, Chamran Heart Center, Isfahan University of Medical Sciences, Isfahan, Iran. *

**Keywords:** *Heart defects, congenital*, *Transposition of great vessels*, *Thoracic surgery*

## Abstract

**Background: **The optimal surgical plan to correct the simple transposition of the great arteries (TGA) is the arterial switch operation (ASO). This study aimed to assess the outcomes of ASO in infants with simple TGA with a special focus on the time of surgery.

**Methods: **This retrospective study enrolled 105 infants with simple TGA who underwent ASO and categorized them into 3 groups based on the time of repair: first week of life: Group A; second week of life: Group B; and third week of life: Group C. The endpoints, comprised of an assessment of pre- and postoperative outcomes, complications, and survival, were compared between the groups.

**Results: **The mean age of the infants was 10.50±6.26 days, and 61 (58.1%) cases were male. The mean number of days on dopamine was 1.85±0.12 in Group A, 2.48±0.03 in Group B, and 2.67±0.08 in Group C (P<0.001). The mean number of days on epinephrine was 1.25±0.07 in Group B and 1.27±0.08 in Group C (P<0.001). The mean number of days on the ventilator was 3.52±0.20 in Group A, 4.56±0.24 in Group B, and 5.06±0.21 in Group C (P<0.001). The mean number of days of ICU stay was 6.69±0.21 in Group A, 8.46±0.57 in Group B, and 9.70±0.64 in Group C (P<0.001). The infants in Group A had a greater survival rate (97.0%) than those in Group B (94.1%) and Group C (78.4%) (P=0.042).

**Conclusion: **ASO in infants with simple TGA can be done within the first week of life with satisfactory outcomes and survival.

## Introduction

Transposition of the great arteries (TGA) is a common and life-threatening congenital heart defect that requires rapid surgery. The optimal surgical plan for simple TGA is the arterial switch operation (ASO), which is commonly performed shortly after birth to prevent left ventricular (LV) deconditioning.^1^^-^^3^ From a clinical point of view, the early diagnosis of TGA is vital to avoid the involution of the LV, which may contribute to the low cardiac output syndrome and LV failure. Assessments of the impact of the surgical time on early outcomes after the repair of TGA have not led to consensus. In spite of the technical limitations of ASO in children with complex TGA, the early and late outcomes following ASO are satisfactory.^4^^, ^^5^ This study aimed to assess the influence of the surgical time on the early outcomes of infants with simple TGA undergoing ASO within the first, second, and third weeks of life.

## Methods

This single-center retrospective study was carried out between 2006 and 2016 on infants with simple TGA who underwent primary ASO. The study protocol was approved by the local ethics committee, and informed consent was obtained from all the individuals participating in the study. 

Early clinical diagnoses had been established in the neonatal period; nonetheless, some infants who had delays between diagnosis and the corrective surgery were transferred to our center from other hospitals. 

The infants were categorized into 3 groups based on the time of repair. Group A consisted of infants who were diagnosed early and underwent primary ASO during the first week of life; Group B was comprised of infants who underwent primary ASO during the second week of life; and Group C comprised infants who were transferred to our center for corrective surgery and underwent primary ASO during the third week of life. Infants with TGA and ventricular septal defect or double-outlet right ventricle and patients who underwent pulmonary artery banding prior to ASO were excluded from this study. 

All the patients underwent standard conventional transthoracic echocardiography and tissue Doppler imaging echocardiography. Most of the study patients had an appropriate biventricular ejection fraction, but the infants in Group C exhibited a slight decrease in the posterior wall thickness. The patent ductus arteriosus of all of the neonates was kept open with E1-prostaglandin (PGE1) preoperatively to improve the systemic oxygen saturation and to maintain a stable hemodynamic balance. Additionally, balloon atrial septostomy was performed in the cases with severe cyanosis to improve blood mixing and prevent arterial desaturation. There were no underlying cardiac disorders in these patients, and they were hemodynamically stable. 

Repair was done with the aid of cardiopulmonary bypass (CPB) under moderate hypothermia. Standard CPB was established with aortic and bicaval cannulation. Cardiac arrest and myocardial protection were induced with a single injection of cold crystalloid cardioplegia and hypothermia (24–28 ^°^C) in all the cases. The ducts arteriosus was ligated before cross-clamping the aorta. With the heart arrested, the aorta and the main pulmonary artery were transected just above the semilunar valves. In all the patients, the LeCompte maneuver was used, where possible. Afterward, the coronary arteries were dissected with a button aortic wall and relocated on to the neoaorta. The defect created by the harvest of the coronary arteries in the native aortic root was repaired with a patch of autologous pericardium. Upon the completion of the repair, CPB was discontinued to allow the resumption of the heart function. In the patients having hemodynamic compromise during the closure of the chest, the sternum was left open until the patient stabilized. 

Postoperative care included the administration of dopamine (5 µg/kg/min) and a low dose of epinephrine (0.03 to 0.3 µg/kg/min) and milrinone (0.65 µg/kg/min) in order to reduce afterload and to maintain adequate peripheral perfusion. Early peritoneal dialysis and extracorporeal membrane oxygenation (ECMO) were used when necessary. Daily postoperative echocardiography was performed during the stay in the intensive care unit (ICU). 

All the statistical analyses were performed using SPSS software (version 22; SPSS Inc., Chicago, IL, USA). The continuous variables are presented as the mean±the standard deviation. One-way ANOVA and the χ^2^ or Fisher exact test were used to find any statistically significant difference between these data. Survival curves were generated by using the Kaplan–Meier survival estimates. *Cox proportional hazards (PH) regression* was employed to identify the risk factors for in-hospital and early mortality. A P<0.05 was considered to be the significant level for all the tests.

## Results

**Figure 1:**
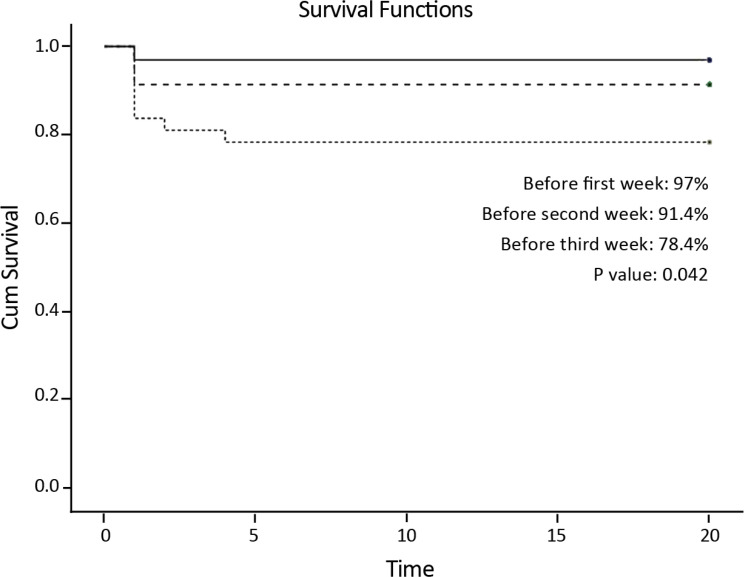
Kaplan–Meier curves of survival after the arterial switch operation for the simple transposition of the great arteries.

Of the120 patients with various kinds of TGA, 105 infants who had simple TGA were enrolled in the current study. The study population was classified as Group A (n=33), Group B (n=35), and Group C (n=37). The patient population consisted of 61 males and 44 females, with a male-to-female ratio of 1/3. The average weight of the patients was 3.78±0.34 kg. Eighty-two infants received preoperative PGE1, and 14 cases underwent balloon atrial septostomy prior to ASO. There were no significant differences between the groups regarding patient characteristics, preoperative care, and intraoperative parameters including CPB and aortic cross-clamp. As is shown in Table 1, the one-way ANOVA analysis revealed a significant difference in inotropic support between the study groups (P<0.001). Moreover, there were significant differences concerning the duration of the ventilation time and the ICU stay between the groups (P<0.001). As is demonstrated in Table 2, there were no significant differences in terms of bleeding, sepsis, neurological disorders, and mortality between the 3 groups. There were 5 cases of mild supra-annular pulmonary stenosis following surgery. Although the infants in Group C had a greater need for ECMO, the difference with the other groups was not statistically significant (P=0.090). Thirteen infants required peritoneal dialysis due to persistent fever not responding to the routine management of fever. Nineteen patients were eligible for delayed sternal closure because of hemodynamic and respiratory instability. The overall mortality rate was 5.7%. There was 1 mortality event in Group A due to acute respiratory distress syndrome. Likewise, 1 infant in Group B died because of sepsis and endocarditis. There were 4 mortality events in Group C, mainly due to acute myocardial failure and LV dysfunction. The Cox PH regression analysis showed that older age, lower body weight, longer aortic cross-clamp time, and lengthier CPB time were the risk factors of early mortality; however, they were not statistically significant (Table 3). The median duration of the clinical follow-up after hospital discharge was 2 years for the survivors. (The medical follow-up data were obtained through direct contact with the referring pediatric cardiologist.) As is depicted in Figure1, the 1-year survival rates in the 3 groups were 97.0%, 94.1%, and 78.4% (P=0.042).

**Table 1:** Comparisons of the pre- and postoperative characteristics between the 3 groups of infants who underwent corrective surgery*

	Group A (n=33)	Group B (n=35)	Group C (n=37)	P
Male/Female	19/14	20/15	22/15	0.978
Weight (kg)	3.67±0.28	3.85±0.30	3.81±0.41	0.151
PG infusion	25 (75.8)	27 (77.1)	30 (81.1)	0.854
Preoperative BAS	1 (3)	5 (14.3)	8 (21.6)	0.072
CPB time (min)	190.27±16.33	182.90±14.01	182.64±8.53	0.332
ACC time (min)	140.36±15.12	142.20±24.80	146.91±8.38	0.658
Days on dopamine	1.85±0.12	2.48±0.08	2.67±0.08	0.001
Days on epinephrine	0	1.25±0.07	1.27±0.08	0.001
Days on ventilator	3.52±0.20	4.56±0.24	5.06±0.21	0.001
ICU stay (d)	6.69±0.21	8.46±0.57	9.70±0.64	0.001

Group A, Corrective surgery in the first week of life; Group B, Corrective surgery in the second week of life; Group C, Corrective surgery in the third week of life; PG, Prostaglandin; BAS, Balloon atrial septostomy; CPB, Cardiopulmonary bypass; ACC, Aortic cross-clamp; ICU, Intensive care unit

* Data are presented as mean±SD or n (%).

**Table 2:** Comparison of the postoperative complications between the 3 groups of infants who underwent corrective surgery*

	Group A (n=33)	Group B (n=35)	Group C (n=37)	P
ECMO	0	1 (2.9)	4 (10.8)	0.090
Bleeding	1 (3.0)	3 (8.6)	3 (8.1)	0.598
Delayed sternal closure	5 (15.2)	7 (20)	7 (18.9)	0.863
Sepsis	0	0	1 (2.7)	0.395
Peritoneal dialysis	3 (9.1)	5 (14.3)	5 (13.5)	0.783
Supra-annular PS	1 (3.0)	2 (5.7)	2 (5.4)	0.851
Neurological disorder	0	1 (2.9)	1 (2.7)	0.626
Mortality	1 (3.0)	1 (2.9)	4 (10.8)	0.252

Group A, Corrective surgery in the first week of life; Group B, Corrective surgery in the second week of life; Group C, Corrective surgery in the third week of life; ECMO, Extracorporeal membrane oxygenation; LV, Left ventricle; PS, Pulmonary stenosis

* Data are presented as n (%).

**Table 3:** Results of the Cox proportional hazard regression analysis of the risk factors for early mortality after ASO

	HR	95% CI	P
Age	1.07	0.56 – 2.02	0.830
Weight	1.10	0.63 – 1.93	0.726
ACC time	1.01	0.98 – 1.01	0.935
CPB time	1.01	0.98 – 1.01	0.907
Intubation time	0.96	0.43 – 2.14	0.939
LV dysfunction	0.90	0.44 – 1.84	0.774
Endocarditis	0.96	0.11 – 7.83	0.974

ASO, Arterial switch operation; HR, Hazard ratio; CI, Confidence interval; ACC, Aortic cross-clamp; CPB, Cardiopulmonary bypass; LV, Left ventricle

P value was analyzed using the log-rank test.

## Discussion

In this single-center retrospective study, the impact of the surgical time on the clinical outcomes of infants with simple TGA undergoing ASO during the first month of life was assessed. Although the surgical time of ASO to repair simple TGA is controversial, our investigation confirms that the best survival and ventricular function outcomes are optimized if ASO is performed during the first 2 weeks of life. 

It has been traditionally plausible that the LV can sustain the systemic pressure up to the age of 21 days. Therefore, ASO is usually proposed for neonates with simple TGA beyond the first few weeks of life due to concern for the ability of the LV to support the systemic circulation. ^6^

In parallel with our results, Kang et al^6^ demonstrated a correlation between an increase in age at surgery time and prolonged intubation and ICU stay time. It is supposed that this condition is a reflection of provisional but reversible LV dysfunction, which is likely to be more noticeable as age increases. It seems reasonable to hypothesize that the LV in older infants with simple TGA might need a longer time to cope with the increased work after ASO. Nonetheless, a wide variation exists in the rate of preoperative LV deconditioning including the size of the interatrial communication and duct, which clearly influences the LV preload and afterload. Moreover, other factors such as a possible genetic predeterminer might play a role in dictating the involution of pulmonary vascular resistance and LV performance.^6^^, ^^7^ Some patients with TGA are recognized to have pulmonary hypertension, while others may continue to have high pulmonary vascular resistance.^8^^, ^^9^ In the presence of high pulmonary blood flow and some degree of precapillary or postcapillary pulmonary hypertension, the LV can grow and keep the capacity to adapt to systemic pressures and remains well conditioned after the first few weeks of life.^10^ An alternative surgical method in these patients is pulmonary artery banding for 1 to 2 weeks before ASO in order to retrain the LV. 

It is suspected that the earliest LV training causes LV remodeling as a result of increased LV afterload, LV wall shear stress, and LV volume overload. This remodeling improves the efficiency of the LV to cope with the systemic circulation. However, the rapid LV retraining approach by pulmonary artery banding yields a less favorable outcome because of high mortality and morbidity rates, prolonged ICU stay, and increased hospital costs compared with primary ASO.^11^^, ^^12^

Additionally, previous investigations have reported that primary ASO is complicated with the high incidence of postoperative right ventricular outflow tract (RVOT) obstruction. Anatomical risk factors in the occurrence of RVOT obstruction after ASO are aortic arch obstruction, the side-by-side position of the great arteries, coronary artery anomalies, and the preoperative existence of subaortic RVOT obstruction. 

However, technical factors such as the insufficient mobilization of the pulmonary arteries and the inadequate size or form of the pericardial patch used to reconstruct the pulmonary trunk have been suggested to contribute to the development of RVOT obstruction.^13^

Numerous studies have shown the success of primary ASO in late presenters with simple TGA. Kang et al.^6^ published their series of primary ASO beyond the age of 21 days, comparing the outcomes with those of ASO up to 21 days. They found only a 3.8% operative mortality rate in the “late switch” group. Ismail et al.^14^ reported a mortality rate of 3.1% in infants who underwent ASO beyond the age of 3 weeks and 7% for infants up to 3 weeks of birth. Davis et al.^15^ reported on 18 infants older than 21 days who underwent primary ASO, with only 1 case of mortality. Duncan et al.^16^ reported on 8 infants aged 15 to 46 days who underwent primary ASO, with no mortality. 

In our series, there was 1 early death related to acute respiratory distress syndrome in infants who underwent ASO during the first week of life. Likewise, there were 1 and 4 mortality events in the infants who underwent ASO, respectively, during the second and third weeks after birth (P=0.252). Our data also confirm previous reports that late ASO performance is associated with a prolonged intubation time, a longer hospital stay, and delayed sternal closure. Meanwhile, older age and lower birth weight in infants with TGA continue to be at increased risk for both early mortality and morbidity after surgery through CPB establishment and complex congenital heart surgery. 

Our results support prior studies showing that late repair of TGA is an important risk factor for death in this population. 

The present study has some limitations. This retrospective investigation enrolled a small number of patients who underwent the surgical repair of simple TGA in a single center. Hence, the statistical analyses may not have sufficient power to support any definitive conclusions. There were also some infants who were referred late from other centers, which may have compromised our study results.

Similar to previous studies, a longer follow-up duration would be also warranted.

## Conclusion

In summary, ASO for patients with simple TGA can still be tolerated beyond the first month of life. According to our results, the best survival and ventricular function outcomes are optimized if ASO is done during the first 2 weeks of life. Furthermore, the surgical outcome and the ICU course are better if ASO is performed in the first week of life. Inasmuch as a decrease in the contractility of the LV and the adaption of the left-side heart with lower pressures in the pulmonary artery can lead to higher mortalities in later weeks, we recommend that simple TGA in neonates be performed during the first week of their life. 

## Notes:

***This paper should be cited as: ***Bigdelian H, Sedighi M. Comparative Outcomes of Primary Arterial Switch Operation for Transposition of Great Arteries within the First Month of Life. J Teh Univ Heart Ctr 2020;15(1):1-5.
